# Recurrent Takotsubo Cardiomyopathy Triggered by COVID-19 Infection Complicated by Ventricular Tachycardia Arrest and Cardiogenic Shock: A Case Report

**DOI:** 10.7759/cureus.110518

**Published:** 2026-06-09

**Authors:** Roberto R Gonzalez Alvarez

**Affiliations:** 1 Internal Medicine, Cape Coral Hospital, Cape Coral, USA

**Keywords:** cardiogenic shock, covid-19, covid-19 and cardiomyopathy, covid-19 associated takotsubo cardiomyopathy, implantable cardioverter-defibrillator (icd), recurrent stress cardiomyopathy, r-on-t phenomenon, takotsubo cardiomyopathy and ventricular arrhythmias, takotsubo cardiomyopathy (tcm), ventricular tachycardia (vt)

## Abstract

Takotsubo cardiomyopathy is a transient stress-induced cardiomyopathy typically triggered by emotional or physical stress. Since the emergence of coronavirus disease 2019 (COVID-19), several cardiovascular complications have been reported, including stress cardiomyopathy. Recurrent Takotsubo cardiomyopathy complicated by malignant ventricular arrhythmias and cardiogenic shock remains uncommon.

A 73-year-old Hispanic female with a prior history of Takotsubo cardiomyopathy in 2013, subclinical hyperthyroidism, and hyperlipidemia presented with worsening malaise and subjective fever. She tested positive for COVID-19. Initial troponin was 0.070 ng/mL, with a peak value of 0.143 ng/mL. Pro-BNP (pro B-type natriuretic peptide) was markedly elevated at 11,250 pg/mL, reflecting significant myocardial wall stress. The initial electrocardiogram showed sinus rhythm with nonspecific T-wave abnormalities in leads III and aVF and a QTc interval of 452 ms. A repeat electrocardiogram six hours later revealed clear T-wave inversions in leads II, III, aVF, and V6. She was transferred to a tertiary care center, where she developed pulseless ventricular tachycardia due to an R-on-T phenomenon, requiring nine minutes of advanced cardiovascular life support. COVID-19 was considered the primary trigger after exclusion of alternative physical stressors. Coronary angiography demonstrated no obstructive coronary artery disease. Right heart catheterization revealed elevated filling pressures and low cardiac output consistent with cardiogenic shock. Transthoracic echocardiography showed an ejection fraction of 10% with apical ballooning typical of Takotsubo cardiomyopathy. Mechanical circulatory support with an intra-aortic balloon pump, inotropes, and vasopressors was required. The patient gradually improved; a repeat echocardiogram on day 9 demonstrated complete normalization of ejection fraction to 70%, cardiac magnetic resonance imaging revealed no late gadolinium enhancement, and sustained recovery was confirmed at outpatient follow-up with an ejection fraction of 60%-70%. Due to the malignant ventricular arrhythmia, a secondary prevention implantable cardioverter-defibrillator was placed. A follow-up electrocardiogram showed sinus rhythm with no ischemic changes, no Q waves, and a QTc of 428 ms.

This case illustrates a recurrent Takotsubo episode temporally associated with COVID-19 infection and complicated by R-on-T ventricular tachycardia and cardiogenic shock. The prior normal myocardial perfusion study and full functional recovery support the diagnosis. A large national database study has shown that COVID-19 patients with stress cardiomyopathy have significantly higher mortality and complication rates. The role of subclinical hyperthyroidism as a potential sensitizer to catecholamine surges is explored. A recently published case of asymptomatic COVID-19 triggering recurrent Takotsubo highlights that the spectrum of this association ranges from subclinical infection to life-threatening presentations.

COVID-19 infection was temporally associated with recurrent Takotsubo cardiomyopathy with life-threatening arrhythmias. Early recognition, aggressive hemodynamic support, and individualized decisions regarding implantable cardioverter-defibrillator placement are critical for favorable outcomes.

## Introduction

Takotsubo cardiomyopathy, also known as stress-induced cardiomyopathy or "broken heart syndrome," is a transient left ventricular systolic dysfunction typically precipitated by emotional or physical stress. The condition characteristically mimics acute coronary syndrome, presenting with chest pain, electrocardiographic changes, and elevated cardiac biomarkers despite the absence of obstructive coronary artery disease, creating a significant diagnostic challenge that often requires coronary angiography for differentiation [1,2]. The syndrome predominantly affects postmenopausal women and accounts for approximately 1%-2% of all patients presenting with suspected acute coronary syndrome [2].

The exact pathophysiology remains incompletely understood; however, catecholamine excess, sympathetic overstimulation, microvascular dysfunction, and myocardial stunning are thought to play major roles [3]. Since the onset of the coronavirus disease 2019 (COVID-19) pandemic, increasing evidence has suggested an association between COVID-19 infection and Takotsubo cardiomyopathy due to systemic inflammation, cytokine storm, endothelial dysfunction, microvascular injury, and stress-mediated catecholamine surges [4,5]. Evidence from Jabri et al. supports a higher incidence of Takotsubo syndrome specifically during acute COVID-19 infection, above and beyond the broader psychosocial stress of the pandemic [4]. A large propensity-matched analysis of the National Inpatient Sample demonstrated that COVID-19 patients with stress cardiomyopathy had significantly higher in-hospital mortality (32.8% vs. 14.6%) and increased rates of mechanical ventilation, vasopressor support, cardiogenic shock, and cardiac arrest compared to those without stress cardiomyopathy [6].

Although most patients experience recovery of ventricular function, severe complications, including cardiogenic shock, ventricular arrhythmias, and cardiac arrest, may occur. Recurrent Takotsubo cardiomyopathy is relatively uncommon, with reported recurrence rates ranging from approximately 1% to 10% in the literature [7]. Recent data from Topf et al. report a recurrence rate of 3.2% in a cohort of 435 patients, with recurrence carrying a 5.9-fold higher risk of 30-day cardiovascular mortality [8]. Ventricular arrhythmias occur in approximately 3%-10% of Takotsubo patients during the acute phase and are associated with increased morbidity and mortality [9]. Recurrent Takotsubo cardiomyopathy associated with COVID-19 infection and complicated by pulseless ventricular tachycardia due to an R-on-T phenomenon remains particularly rare.

We present a case of recurrent Takotsubo cardiomyopathy triggered by COVID-19 infection in an elderly female, complicated by ventricular tachycardia arrest and cardiogenic shock requiring mechanical circulatory support.

## Case presentation

A 73-year-old Hispanic woman with a prior history of Takotsubo cardiomyopathy diagnosed in 2013, subclinical hyperthyroidism treated with methimazole 2.5 mg daily, multinodular goiter, hyperlipidemia, and benign colonic polyps presented to the emergency department in November 2022 with two days of worsening malaise, subjective fever, headache, nonproductive cough, generalized body aches, anosmia, and decreased appetite. She denied chest pain, dyspnea, palpitations, or syncope.

On arrival, vital signs were blood pressure 150/89 mmHg, heart rate 82 beats/min, respiratory rate 18/min, temperature 98.4°F, and oxygen saturation 100% on ambient air. Initial laboratory studies at 04:49 hours revealed a high-sensitivity troponin of 0.070 ng/mL (reference range: 0.000-0.010 ng/mL) and a positive polymerase chain reaction test for SARS-CoV-2. Pro BNP (pro B-type natriuretic peptide) was markedly elevated at 11,250 pg/mL (reference range: 1-125 pg/mL), reflecting significant myocardial wall stress. C-reactive protein was elevated at 10.7 mg/L (reference range: 0.0-5.0 mg/L), and ferritin was mildly elevated at 178 ng/mL (reference range: 15-150 ng/mL). D-dimer was normal (<150 ng/dL; reference range: <365 ng/dL), procalcitonin was normal (0.050 ng/mL; reference range: 0.020-0.080 ng/mL), lactate dehydrogenase (LDH) was normal (194 U/L; reference range: 135-214 U/L), and creatine phosphokinase (CPK) was normal (97 U/L; reference range: 20-180 U/L). Complete blood count and basic metabolic panel were within normal limits. The initial electrocardiogram (EKG) showed normal sinus rhythm with a nonspecific T-wave abnormality, most pronounced as T-wave inversion in leads III and aVF (deeper in III), with normal T-waves in leads II and V6 and a QTc interval of 452 ms (Figure 1). Chest radiography demonstrated bilateral lower-lobe atelectatic changes. Computed tomography of the head revealed age-related atrophy and no acute pathology.

**Figure 1:**
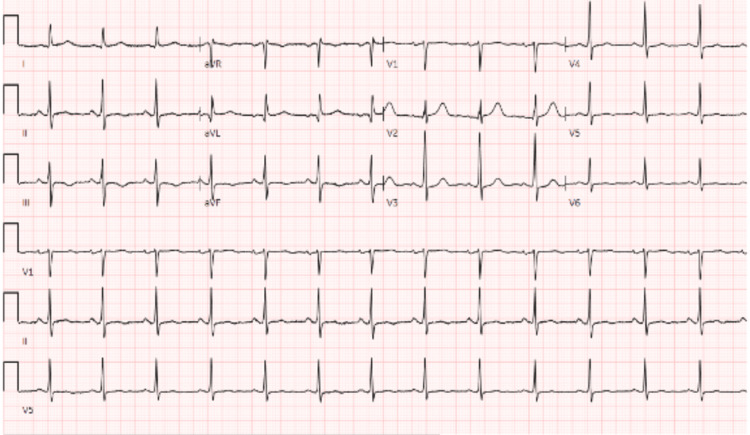
Initial Electrocardiogram on Presentation (November 26, 2022) Twelve-lead electrocardiogram obtained in the emergency department demonstrating normal sinus rhythm with nonspecific T-wave abnormality, most pronounced as T-wave inversion in leads III and aVF (deeper in III), with normal T-waves in leads II and V6. QTc interval: 452 ms.

She was given acetaminophen and aspirin and was admitted for telemetry monitoring. Repeat troponin at 11:13 hours increased to 0.143 ng/mL, representing the peak value, and a repeat EKG demonstrated new T-wave inversions now clearly present in leads II, III, aVF, and V6 (Figure 2). She remained chest pain-free but had one episode of vomiting. Cardiac care was consulted; a loading dose of clopidogrel and intravenous heparin were administered for suspected acute coronary syndrome. The patient was transferred to a tertiary care center (Bellevue Hospital) for further management.

**Figure 2:**
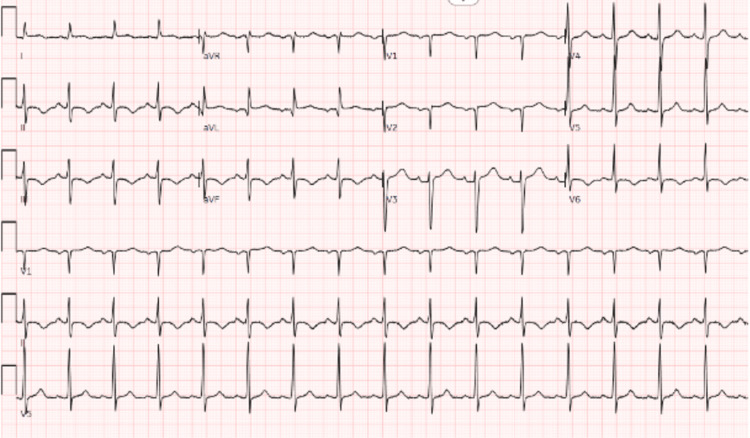
Repeat Electrocardiogram (November 26, 2022) Repeat electrocardiogram performed approximately six hours after presentation. New T-wave inversions are now clearly present in leads II, III, aVF, and V6, consistent with inferolateral myocardial injury.

On the second hospital day, the patient developed ventricular tachycardia, documented on a pre-arrest EKG showing wide-complex ventricular tachycardia across all leads and derivations (Figure 3). The R-on-T phenomenon was documented on telemetry review by the cardiology team, which showed a premature ventricular complex falling on the descending limb of the T wave immediately preceding the polymorphic ventricular tachycardia. This rhythm was attributed to an R-on-T phenomenon and rapidly degenerated into pulseless ventricular tachycardia. Return of spontaneous circulation was achieved after nine minutes of advanced cardiovascular life support. She was intubated and taken emergently to the cardiac catheterization laboratory. Coronary angiography revealed no obstructive coronary artery disease. Right heart catheterization showed elevated filling pressures with a pulmonary capillary wedge pressure of 22 mmHg and a low cardiac index of 1.8 L/min/m², consistent with cardiogenic shock. Transthoracic echocardiography performed on November 27 demonstrated severe left ventricular systolic dysfunction with an ejection fraction (EF) of 10%, apical akinesis with preserved contractility in the base, apical thinning, and spontaneous echocardiographic contrast ("smoke") in the left ventricle, findings consistent with the apical ballooning pattern typical of Takotsubo cardiomyopathy. The left atrium and right atrium were mildly enlarged. There was no significant valvular disease and no pericardial effusion. Left ventricular outflow tract obstruction was not formally assessed on this emergent study. An intra-aortic balloon pump (IABP) was placed, and vasopressor and inotrope infusions were initiated, including norepinephrine at an initial dose of 0.1 mcg/kg/min, titrated to a mean arterial pressure of ≥65 mmHg, and dobutamine at an initial dose of 5 mcg/kg/min. 

**Figure 3:**
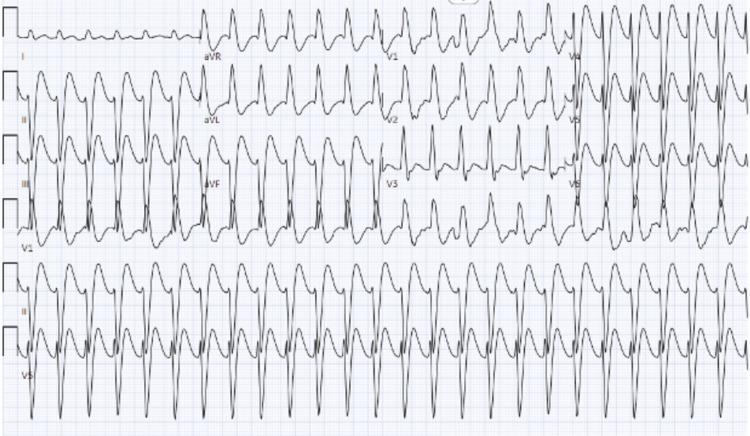
Pre-arrest Electrocardiogram (November 27, 2022) Electrocardiogram obtained immediately prior to cardiac arrest demonstrating wide-complex ventricular tachycardia across all leads and derivations, consistent with pulseless ventricular tachycardia triggered by an R-on-T phenomenon. QTc cannot be reliably calculated from this tracing due to the wide-complex rhythm.

Over the subsequent days, her hemodynamics gradually improved. The IABP was removed on hospital day 4, and all pressors and inotropes were discontinued by day 6. Her course was complicated by transient liver enzyme elevation (alkaline phosphatase peaking at 289 U/L, gamma-glutamyl transferase 689 U/L) attributed to shock liver, which resolved spontaneously. An abdominal ultrasound demonstrated only biliary sludge. The patient was intubated on November 27 and was successfully extubated on December 2, representing a total of five days of invasive mechanical ventilation. She was transferred to the step-down unit. Neurological evaluation after cardiac arrest confirmed full neurological recovery with no focal deficits; the patient was following commands appropriately after extubation.

A repeat transthoracic echocardiogram performed on December 6 demonstrated complete normalization of left ventricular systolic function with an EF of 70% and normal wall motion, indicating full recovery within nine days of the initial insult. A trace pericardial effusion was noted. The left ventricle was normal in size with normal wall thickness and normal diastolic function. Right ventricular size and function were normal. There was minimal mitral regurgitation, minimal tricuspid regurgitation, and minimal pulmonic regurgitation. The aortic valve was sclerotic without stenosis.

Cardiac magnetic resonance imaging performed on December 13 revealed improvement in EF to 44% without late gadolinium enhancement, which is supportive of the diagnosis of Takotsubo cardiomyopathy, although it does not completely exclude myocarditis, particularly given that imaging was performed during partial recovery. Given the documented malignant ventricular arrhythmia, a secondary prevention implantable cardioverter-defibrillator (ICD) was placed on December 14. By discharge on December 16, the patient was ambulating with assistance and was on a regimen of carvedilol 12.5 mg twice daily, losartan 25 mg daily, empagliflozin 10 mg daily, and methimazole 2.5 mg daily. Empagliflozin was initiated as part of guideline-directed medical therapy for heart failure with reduced EF, consistent with current recommendations to initiate SGLT2 (sodium-glucose cotransporter 2) inhibitors in patients with left ventricular dysfunction regardless of etiology, with the goal of providing comprehensive neurohormonal blockade during the recovery period.

Of note, a nuclear myocardial perfusion stress study performed on January 14, 2022, 10 months before the current admission, had shown a normal EF of 70% and no ischemic perfusion defects, documenting complete recovery from the 2013 episode. This study was ordered as part of routine outpatient cardiology follow-up approximately nine years after the initial Takotsubo episode; the patient was asymptomatic from a cardiac standpoint at the time, with no chest pain or dyspnea.

At outpatient follow-up on January 6, 2023, the patient was clinically stable, normotensive, and slightly hypovolemic. A follow-up EKG demonstrated sinus rhythm with no ischemic changes, no pathologic Q waves, and a QTc interval of 428 ms (Figure 4). Repeat echocardiography revealed an EF of 60%-70%, confirming sustained recovery. Guideline-directed medical therapy was adjusted due to dizziness. She continues to do well. 

**Figure 4:**
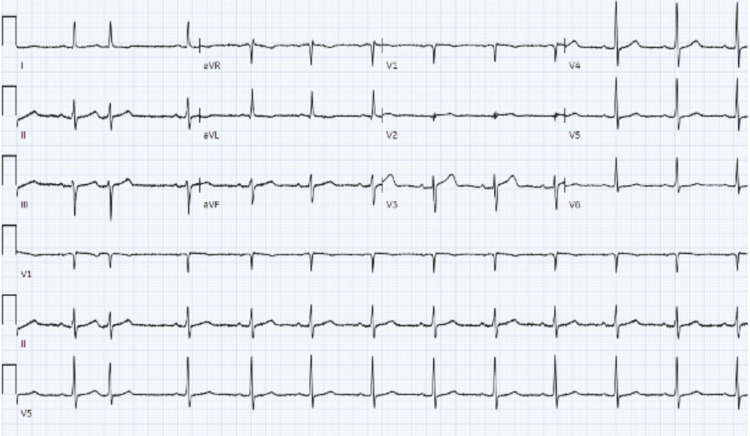
Follow-Up Electrocardiogram (January 6, 2023) Outpatient follow-up electrocardiogram showing sinus rhythm with no ischemic changes and no pathologic Q waves, indicating complete electrocardiographic recovery. QTc interval: 428 ms.

Key laboratory findings are summarized in Table 1. The timeline of hospitalization and major clinical events is presented in Figure 5. The patient met the InterTAK diagnostic criteria for Takotsubo syndrome (Table 2). 

**Figure 5:**
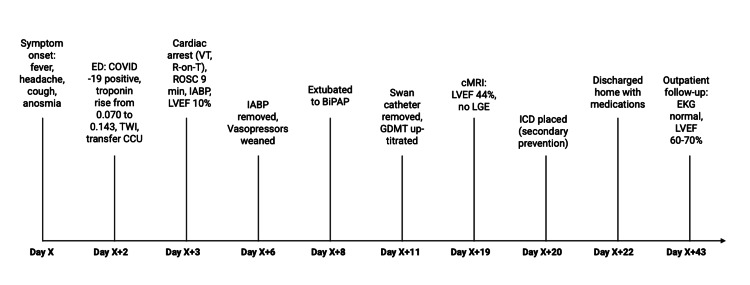
Timeline of Hospitalization and Major Clinical Events Timeline illustrating key clinical events from symptom onset through outpatient follow-up in a 73-year-old woman with recurrent Takotsubo cardiomyopathy triggered by COVID-19 infection. Day X denotes the date of symptom onset. Subsequent time points are expressed as days after symptom onset. ED, emergency department; COVID+, coronavirus disease 2019 positive; trop, troponin; TWI, T-wave inversions; CCU, cardiac care unit; VT, ventricular tachycardia; R‑on‑T, R‑on‑T phenomenon; ROSC, return of spontaneous circulation; IABP, intra‑aortic balloon pump; LVEF, left ventricular ejection fraction; BiPAP, bilevel positive airway pressure; GDMT, guideline‑directed medical therapy; cMRI, cardiac magnetic resonance imaging; LGE, late gadolinium enhancement; ICD, implantable cardioverter‑defibrillator. This is an original figure created by the authors using Microsoft PowerPoint  (Microsoft Corporation, Redmond, Washington) and Adobe Illustrator (Adobe Inc., San Jose, California). No AI-generated tools were used. No permission is required.

**Table 1:** Key Laboratory and Diagnostic Findings During Hospitalization Pro BNP, pro B-type natriuretic peptide; CPK, creatine phosphokinase; CRP, C-reactive protein; LDH, lactate dehydrogenase; LVEF, left ventricular ejection fraction; PCWP, pulmonary capillary wedge pressure; GGT, gamma-glutamyl transferase; CBC, complete blood count; BMP, basic metabolic panel; LGE, late gadolinium enhancement; NM, nuclear medicine; SPECT, single-photon emission computed tomography.

Parameter	Value	Reference Range	Date/Time
Cardiac biomarkers			
Troponin (ng/mL), initial	0.07	0.000-0.010	11/26/22 4:49
Troponin (ng/mL), peak	0.143	0.000-0.010	11/26/22 11:13
Troponin (ng/mL), follow-up	0.059	0.000-0.010	12/3/22
Pro BNP (pg/mL)	11,250	1-125	11/26/22
CPK (U/L)	97	20-180	11/26/22
Inflammatory and infection markers			
CRP (mg/L)	10.7	0.0-5.0	11/26/22
Ferritin (ng/mL)	178	15-150	11/26/22
Procalcitonin (ng/mL)	0.05	0.020-0.080	11/26/22
LDH (U/L)	194	135-214	11/26/22
D-dimer (ng/dL)	<150	<365	11/26/22
COVID-19 PCR	Positive	Negative	11/26/22
Electrocardiographic parameters			
QTc interval (ms), admission	452	<440	11/26/22
QTc interval (ms), follow-up	428	<440	1/6/23
Echocardiographic parameters			
LVEF (%), nadir	10	≥55	11/27/22
LVEF (%), recovery	70	≥55	12/6/22
LVEF (%), follow-up	60-70	≥55	1/6/23
Hemodynamic parameters (right heart catheterization)			
Cardiac index (L/min/m²)	1.8	2.5-4.0	11/27/22
PCWP (mmHg)	22	6-12	11/27/22
Other laboratory parameters			
Alkaline phosphatase (U/L), initial	71	44-147	11/26/22
Alkaline phosphatase (U/L), peak	289	44-147	12/2/22
GGT (U/L), peak	689	5-40	12/2/22
CBC	Within normal limits	—	11/26/22
BMP	Within normal limits	—	11/26/22
Cardiac imaging			
Cardiac MRI	EF 44%, no LGE	—	12/13/22
NM perfusion SPECT (1/2022)	EF 70%, no ischemia	—	1/14/22

**Table 2:** InterTAK Diagnostic Criteria Applied to the Present Case Adapted from Ghadri JR, Wittstein IS, Prasad A, et al. International Expert Consensus Document on Takotsubo Syndrome (Part I): Clinical Characteristics, Diagnostic Criteria, and Pathophysiology. *Eur Heart J*. 2018;39:2032-46. This table was created by the authors based on the published criteria (Creative Commons CC-BY-NC); no permission is required.

Criterion	Present Case
1. Transient regional wall motion abnormalities of LV or RV (hypokinesia, akinesia, or dyskinesia) presenting as apical ballooning or midventricular, basal, or focal wall motion abnormalities; usually extends beyond a single epicardial vascular distribution	Apical akinesis with preserved basal contractility and apical ballooning pattern on echocardiography with EF 10%; wall motion abnormality beyond a single coronary territory; complete recovery to EF 70% by day 9
2. An emotional, physical, or combined trigger can precede the event, but this is not obligatory	COVID-19 infection (physical trigger)
3. Neurologic disorders or pheochromocytoma may serve as triggers	None present
4. New ECG abnormalities (ST-segment elevation, ST-segment depression, T-wave inversion, QTc prolongation)	Nonspecific T-wave abnormality (III, aVF) progressing to clear T-wave inversions in II, III, aVF, and V6; QTc 452 ms on admission, not meeting criteria for marked prolongation
5. Moderate elevation in cardiac biomarkers (troponin, CK); significant elevation of natriuretic peptide is common	Troponin elevated (peak 0.143 ng/mL); Pro BNP markedly elevated at 11,250 pg/mL, consistent with significant myocardial wall stress
6. Significant coronary artery disease is not a contradiction	Coronary angiography showed no obstructive disease
7. No evidence of infectious myocarditis	Cardiac MRI showed no late gadolinium enhancement; this finding is supportive of rather than absolutely exclusionary for myocarditis. Combined with the clinical course, absence of LGE makes myocarditis less likely.
8. Postmenopausal women are predominantly affected	73-year-old postmenopausal female

Note that echocardiographic and cardiac MRI images were not available for publication. The findings described in the manuscript are based on official cardiology reports.

## Discussion

Takotsubo cardiomyopathy accounts for approximately 1%-2% of all patients presenting with suspected acute coronary syndrome and predominantly affects postmenopausal women [1,2]. Since the onset of the COVID-19 pandemic, an increasing number of stress cardiomyopathy cases have been linked to SARS-CoV-2 infection [4,5]. A systematic review by Ghasemi et al. identified 102 cases of Takotsubo syndrome associated with COVID-19, with a mean left ventricular EF of 36.5% and an in-hospital mortality rate of 33.3% [10]. Importantly, a propensity-matched analysis of the National Inpatient Sample by Davis et al. showed that COVID-19 patients with stress cardiomyopathy had a more than two-fold increase in in-hospital mortality (adjusted OR 2.3) and significantly higher rates of cardiogenic shock, cardiac arrest, and mechanical ventilation [6]. The present case adds to this growing body of literature but is distinguished by several critical features: confirmed recurrence after nine years, a malignant ventricular arrhythmia mediated by the R-on-T phenomenon, a remarkably rapid echocardiographic recovery within nine days, and the complex interplay with subclinical hyperthyroidism.

Recurrence

The recurrence rate of Takotsubo cardiomyopathy is estimated at 1%-10%, with most studies reporting rates around 4% over long-term follow-up [7,11]. Topf et al. recently reported a recurrence rate of 3.2% in a cohort of 435 patients, with recurrence carrying a 5.9-fold higher risk of 30-day cardiovascular mortality (21.4% vs. 3.8%), underscoring the need for closer surveillance in this population [8]. Recurrences are typically triggered by a different emotional or physical stressor than the index event. Our patient's first episode in 2013 was presumably also stress-mediated, and the 2022 recurrence was temporally associated with COVID-19 infection. Importantly, a nuclear myocardial perfusion study performed in January 2022, just 10 months before the current admission, demonstrated a normal left ventricular EF of 70% and no ischemic perfusion defects. This confirms that the ventricle had fully recovered structurally and functionally from the initial insult, making the recurrent episode a genuine de novo stress cardiomyopathy rather than an exacerbation of chronic dysfunction. Recurrent cases remain rare enough that each one offers an opportunity to understand predisposing factors and long-term management.

Echocardiographic findings and rapid recovery

The echocardiographic findings in this case warrant specific attention. The initial study on November 27 revealed classic features of Takotsubo cardiomyopathy: severe left ventricular systolic dysfunction with an EF of 10%, apical akinesis with preserved contractility in the base, apical thinning, and spontaneous echocardiographic contrast ("smoke") in the left ventricle. The presence of spontaneous echocardiographic contrast indicates a low-flow state and blood stasis within the left ventricular cavity, which can predispose to thrombus formation and represents a recognized complication of severe apical ballooning. This finding supports the use of anticoagulation in selected patients with severe Takotsubo cardiomyopathy, although our patient was already receiving heparin for suspected acute coronary syndrome at the time. Remarkably, a repeat echocardiogram just nine days later (December 6) demonstrated complete normalization of left ventricular systolic function with an EF of 70% and normal wall motion. This exceptionally rapid recovery (among the fastest documented in the literature) is characteristic of Takotsubo cardiomyopathy and reinforces the diagnosis. A trace pericardial effusion noted on the recovery study is a common and typically self-limited finding in stress cardiomyopathy.

Electrocardiographic evolution

The electrocardiographic evolution in this case merits specific attention. The initial EKG demonstrated only a nonspecific T-wave abnormality confined to leads III and aVF, which could easily be dismissed as clinically insignificant in a patient without chest pain. However, within six hours, clear T-wave inversions emerged in leads II, III, aVF, and V6, signaling an inferolateral pattern of myocardial injury. The admission QTc was 452 ms, which is mildly prolonged but did not meet criteria for marked QT prolongation. The follow-up QTc normalized to 428 ms, consistent with the resolution of repolarization abnormalities. This rapid progression from subtle to overt repolarization abnormalities underscores the importance of serial electrocardiography in patients with COVID-19 and elevated cardiac biomarkers, even in the absence of typical anginal symptoms. However, this observation is based on a single case and may not represent a generalizable early warning sign. The subsequent pre-arrest EKG captured the exact moment of transition from sinus rhythm to wide-complex ventricular tachycardia across all leads, documenting the electrophysiological substrate for the R-on-T phenomenon.

Ventricular arrhythmia and the R-on-T phenomenon

Ventricular arrhythmias occur in approximately 3%-10% of Takotsubo patients during the acute phase [9]. The mechanism is often multifactorial, including QT-interval prolongation, catecholamine-mediated myocyte calcium overload, and repolarization dispersion. In this patient, the R-on-T phenomenon was documented on telemetry review by the cardiology team, showing a premature ventricular complex falling on the descending limb of the T wave immediately preceding the polymorphic ventricular tachycardia. Although the specific rhythm strip is not available for publication, the documentation supports the electrophysiological mechanism described. This is a well-recognized electrophysiological trigger for Torsades de pointes and polymorphic ventricular tachycardia in the setting of abnormal repolarization [12]. The R-on-T phenomenon occurs when a premature ventricular complex occurs during the vulnerable period of ventricular repolarization, which can, in turn, produce an extrasystole capable of initiating ventricular fibrillation. The admission QTc of 452 ms, while only mildly prolonged, may have contributed to the substrate for this event, as dynamic changes in repolarization during acute stress cardiomyopathy can predispose to such arrhythmias. The successful resuscitation and full neurologic recovery underscore the importance of continuous telemetry and prompt defibrillation in the acute phase.

Alternative diagnoses

Several alternative diagnoses were considered and are important to address. COVID-19 myocarditis was considered less likely given the characteristic apical ballooning pattern on echocardiography (rather than diffuse or patchy dysfunction), the absence of late gadolinium enhancement on cardiac MRI, and the rapid recovery of ventricular function. Inflammatory markers were modestly elevated (C-reactive protein (CRP) 10.7 mg/L, ferritin 178 ng/mL), with normal procalcitonin (0.050 ng/mL) and LDH (194 U/L), findings consistent with COVID-19-related systemic inflammation rather than severe hyperinflammatory syndrome or bacterial superinfection. However, we acknowledge that the absence of late gadolinium enhancement does not definitively exclude myocarditis, particularly because cardiac MRI was performed during partial recovery (EF 44%), which may reduce sensitivity for detecting myocardial inflammation. Myocardial infarction with non-obstructive coronary arteries (MINOCA) was considered but was less likely given the regional wall motion abnormality extending beyond a single epicardial coronary territory, the classic apical ballooning pattern, and complete functional recovery without evidence of infarction on cardiac MRI. Demand ischemia was considered unlikely because the patient was hemodynamically stable at presentation with only modest troponin elevation, and the subsequent deterioration was arrhythmia-mediated rather than driven by ongoing ischemia.

Role of subclinical hyperthyroidism

The patient's history of subclinical hyperthyroidism (on methimazole 2.5 mg daily) deserves attention. Thyroid hormones increase myocardial sensitivity to circulating catecholamines by upregulating beta-adrenergic receptors and enhancing sympathetic tone [13]. Hyperthyroidism, even in its subclinical form, is an independent risk factor for Takotsubo cardiomyopathy and has been reported to lower the threshold for stress-induced myocardial stunning [14,15]. In a comprehensive review, it was found that thyroid disorders were present in 43.4% of Takotsubo cases associated with endocrine conditions [16]. In this case, the combination of COVID-19-related cytokine surge and hyperthyroid catecholamine sensitization may have synergistically precipitated the severe clinical picture. The patient met the InterTAK diagnostic criteria for Takotsubo syndrome, as summarized in Table 2 [17]. Although methimazole was continued, thyroid function testing during hospitalization would have been informative. Regardless, awareness of this association is crucial for clinicians managing patients with overlapping endocrine and cardiovascular pathology. We acknowledge that the contribution of subclinical hyperthyroidism, while plausible, remains speculative without confirmatory thyroid function testing during the acute episode.

ICD decision

The indication for an ICD in Takotsubo cardiomyopathy remains controversial. Because left ventricular dysfunction is typically transient, guideline-directed medical therapy and watchful waiting are the standard for primary prevention. Alternative management strategies could have been considered, including prolonged rhythm monitoring or a temporary wearable cardioverter-defibrillator. However, the treating team opted for a permanent ICD given the documented malignant arrhythmia, the recurrent nature of the patient's Takotsubo syndrome, and the potential for ongoing catecholamine sensitivity. This decision was individualized, and the long-term benefit of ICD therapy in this population remains uncertain. The European Society of Cardiology notes that, in view of the transient nature of Takotsubo syndrome, implantation of a cardioverter-defibrillator for secondary prevention of ventricular arrhythmias may not be required and should be considered on a case-by-case basis [9]. However, in patients who survive a documented malignant arrhythmia, secondary prevention ICD implantation is recommended by current electrophysiology guidelines [18]. The decision in this case was individualized: the patient had a documented pulseless ventricular tachycardia arrest in the setting of acute myocardial stunning. Although her EF recovered, the residual risk of recurrence was considered meaningful given her history of recurrent Takotsubo syndrome and possible ongoing catecholamine sensitivity. Case reports by El-Battrawy et al. have similarly suggested the benefit of ICD insertion, regardless of improvement in left ventricular function, in patients with recurrent Takotsubo syndrome who present with life-threatening arrhythmias [19]. The heart team's decision to proceed with ICD placement on December 14 was therefore reasonable and serves as a teaching point regarding risk stratification in this population.

Cardiac MRI and recovery discrepancy

The temporary discrepancy between the cardiac MRI (EF 44% on December 13) and the echocardiogram performed one week earlier (EF 70% on December 6) warrants discussion. MRI was performed while the patient was relatively early in the recovery phase; furthermore, different imaging modalities can yield slightly different EF values. The absence of late gadolinium enhancement on MRI supports the diagnosis of Takotsubo cardiomyopathy, although it does not completely exclude myocarditis, particularly given that imaging was performed after partial recovery of ventricular function. The echocardiographic documentation of complete recovery to an EF of 70% by December 6, prior to the MRI, indicates that functional recovery was already complete by the time the MRI was obtained. The lower EF on MRI may reflect modality-specific differences in quantification rather than a true discrepancy. Sustained recovery was confirmed at outpatient follow-up in January 2023 (EF 60%-70%). This highlights the utility of cardiac MRI in the diagnostic workup of Takotsubo syndrome, particularly for excluding myocarditis and myocardial infarction, while acknowledging its limitations [20].

Comparison with published literature

Table 3 presents a comparison of our case with selected published reports of recurrent Takotsubo associated with COVID-19 or complicated by ventricular arrhythmia. While isolated COVID-19-associated stress cardiomyopathy is now well documented, the combination of prior confirmed Takotsubo syndrome, R-on-T-triggered cardiac arrest, rapid nine-day echocardiographic recovery, and cardiogenic shock requiring mechanical support appears to be rare. We have also included a case by Patel et al. (2016) describing recurrent Takotsubo related to thyrotoxicosis without COVID-19, to better contextualize ICD decision-making in non-COVID settings [12]. Notably, Dall'Ara et al. recently reported a case of asymptomatic SARS-CoV-2 infection triggering recurrent Takotsubo syndrome, highlighting that even subclinical COVID-19 can serve as a trigger in susceptible patients [21]. In contrast, our patient presented with symptomatic COVID-19 and developed a far more severe clinical course, including R-on-T ventricular tachycardia arrest and cardiogenic shock requiring intra-aortic balloon pump support. Together, these cases illustrate that the spectrum of COVID-19-associated recurrent Takotsubo syndrome ranges from asymptomatic infection with uncomplicated recovery to life-threatening presentations requiring advanced mechanical circulatory support. This case reinforces the need for heightened clinical vigilance in postmenopausal women with a history of stress cardiomyopathy who present with SARS-CoV-2 infection. 

**Table 3:** Reported Cases of Recurrent Takotsubo Cardiomyopathy Associated With COVID-19 or Complicated by Malignant Ventricular Arrhythmia The comparison cases are representative examples identified through a PubMed search. This table is original and created by the authors for this manuscript. The Lu et al. (2024) row represents aggregated data from a systematic review rather than an individual case; it is included to demonstrate the broader literature context. TCM, Takotsubo cardiomyopathy; MCS, mechanical circulatory support; EF, ejection fraction; ICD, implantable cardioverter-defibrillator; IABP, intra-aortic balloon pump; VT, ventricular tachycardia; VF, ventricular fibrillation.

Study	Age/Sex	Prior TCM	Trigger	Arrhythmia	Cardiogenic Shock/MCS	Nadir EF → Recovery EF	ICD	Key Difference From Our Case
Sattar et al. (2021) [22]	68/F	Yes (5 years prior)	COVID-19	None	No	25% → 60%	No	No malignant arrhythmia; milder course
Lu et al. (2024) [23]	— (systematic review)	Mixed	COVID-19	Mixed	Mixed	Mixed	Mixed	Aggregate data; no single case with all features
El-Battrawy et al. (2019) [19]	65/F	Yes (3 years prior)	Emotional	VF arrest	Yes/vasopressors only	20% → 65%	Yes (secondary prevention)	Trigger not COVID-19; no mechanical support
Dall'Ara et al. (2024) [21]	74/F	Yes (2 years prior)	COVID-19 (asymptomatic)	None	No	Not specified → Normal	No	Asymptomatic COVID-19; no arrhythmia
Patel et al. (2016) [12]	63/F	Yes (recurrent)	Thyrotoxicosis	None	No	Not specified → Normal	No	Recurrent TCM with endocrine trigger; no COVID-19; no malignant arrhythmia; included to contextualize ICD decision-making in non-COVID settings
Present case (2024)	73/F	Yes (9 years prior)	COVID-19	Pulseless VT (R-on-T)	Yes/IABP + inotropes	10% → 70%	Yes (secondary prevention)	Recurrent TCM, R-on-T, IABP, ICD; rapid 9-day recovery

Limitations

This case report has several limitations that should be acknowledged. First, echocardiographic and cardiac MRI images were not available for publication; the imaging findings are based on official cardiology reports. Second, the rhythm strip documenting the R-on-T phenomenon was not retained for publication, and our attribution of the arrhythmia mechanism relies on the cardiology team's documentation. Third, although Pro BNP, CRP, ferritin, D-dimer, LDH, and procalcitonin were measured and are reported, interleukin-6, erythrocyte sedimentation rate, serial cardiac biomarkers, and thyroid function tests were not obtained during the acute hospitalization, which limits our ability to fully characterize the inflammatory response and the role of hyperthyroidism. Fourth, left ventricular outflow tract obstruction was not formally assessed on the initial echocardiogram, which may have influenced management decisions regarding inotropes and the IABP. Fifth, as a single case report, our findings cannot establish causality between COVID-19 infection and Takotsubo recurrence, and the generalizability of our observations, particularly regarding the electrocardiographic progression and ICD decision-making, is inherently limited. Finally, the absence of long-term follow-up data beyond the January 2023 visit limits our ability to comment on the durability of ventricular function recovery and the long-term appropriateness of ICD therapy.

## Conclusions

COVID-19 infection was temporally associated with recurrent Takotsubo cardiomyopathy with severe complications, including ventricular tachycardia arrest and cardiogenic shock. This case demonstrates a notable electrocardiographic progression from subtle nonspecific T-wave abnormalities to overt inferolateral T-wave inversions, although this observation is based on a single case and may not represent a generalizable early warning sign. The remarkably rapid echocardiographic recovery from an EF of 10% to 70% within nine days underscores the transient nature of Takotsubo cardiomyopathy and reinforces the importance of serial imaging. The R-on-T phenomenon serves as a specific arrhythmia mechanism in the setting of repolarization abnormalities. The potential role of subclinical hyperthyroidism as a catecholamine sensitizer lowering the threshold for recurrence, while plausible, remains speculative. Decisions regarding ICD implantation in Takotsubo syndrome should remain individualized because the long-term benefit remains uncertain, and alternative strategies such as prolonged rhythm monitoring or temporary wearable cardioverter-defibrillators warrant consideration. The utility of cardiac MRI in supporting the diagnosis by demonstrating the absence of late gadolinium enhancement is recognized, while acknowledging that this finding does not completely exclude myocarditis. Early recognition, prompt hemodynamic support, and multidisciplinary management are critical to improving outcomes in these patients. Future research should address unresolved questions regarding optimal arrhythmia prevention strategies after recovery of ventricular function, including the potential role of wearable cardioverter-defibrillators, prolonged rhythm monitoring, and risk stratification tools to identify patients at the highest risk for recurrent malignant arrhythmias. This case additionally highlights the potential for significant recovery of ventricular function despite severe initial presentation and underscores the importance of considering Takotsubo cardiomyopathy in elderly women presenting with elevated cardiac biomarkers and acute cardiac dysfunction during COVID-19 infection.
